# Lamina propria macrophage phenotypes in relation to *Escherichia coli* in Crohn’s disease

**DOI:** 10.1186/s12876-015-0305-3

**Published:** 2015-07-03

**Authors:** Timothy R. Elliott, Neil B. Rayment, Barry N. Hudspith, Rebecca E. Hands, Kirstin Taylor, Gareth C. Parkes, Natalie J. Prescott, Liljana Petrovska, John Hermon-Taylor, Jonathan Brostoff, Alex Boussioutas, Christopher G. Mathew, Stephen A. Bustin, Jeremy D. Sanderson

**Affiliations:** 1 Diabetes and Nutritional Sciences Division, King’s College London, Franklin Wilkins Building, 150 Stamford Street, London, SE1 9NH UK; 2 Department of Gastroenterology, Guy’s and St Thomas’ NHS Foundation Trust, St Thomas’ Hospital, London, SE1 7EH UK; 3 Department of Medical and Molecular Genetics, King’s College London School of Medicine, Guy’s Hospital, London, SE1 9RT UK; 4 Blizard Institute, Barts and the London School of Medicine and Dentistry, Queen Mary University of London, Whitechapel, London, E1 1BB UK; 5 Department of Medicine, University of Melbourne, Melbourne, Australia; 6 Postgraduate Medical Institute, Faculty of Medical Science, Anglia Ruskin University, Chelmsford, Essex UK

**Keywords:** Crohn’s disease, *Escherichia coli*, Macrophages

## Abstract

**Background:**

Abnormal handling of *E. coli* by lamina propria (LP) macrophages may contribute to Crohn’s disease (CD) pathogenesis. We aimed to determine LP macrophage phenotypes in CD, ulcerative colitis (UC) and healthy controls (HC), and in CD, to compare macrophage phenotypes according to *E. coli* carriage.

**Methods:**

Mucosal biopsies were taken from 35 patients with CD, 9 with UC and 18 HCs. Laser capture microdissection was used to isolate *E. coli*-laden and unladen LP macrophages from ileal or colonic biopsies. From these macrophages, mRNA was extracted and cytokine and activation marker expression measured using RT-qPCR.

**Results:**

*E. coli*-laden LP macrophages were identified commonly in mucosal biopsies from CD patients (25/35, 71 %), rarely in UC (1/9, 11 %) and not at all in healthy controls (0/18). LP macrophage cytokine mRNA expression was greater in CD and UC than healthy controls. In CD, *E. coli*-laden macrophages expressed high IL-10 & CD163 and lower TNFα, IL-23 & iNOS irrespective of macroscopic inflammation. In inflamed tissue, *E. coli*-unladen macrophages expressed high TNFα, IL-23 & iNOS and lower IL-10 & CD163. In uninflamed tissue, unladen macrophages had low cytokine mRNA expression, closer to that of healthy controls.

**Conclusion:**

In CD, intra-macrophage *E. coli* are commonly found and LP macrophages express characteristic cytokine mRNA profiles according to *E. coli* carriage. Persistence of *E. coli* within LP macrophages may provide a stimulus for chronic inflammation.

## Background

The exact pathogenesis of Crohn’s disease (CD) remains unclear, but it is likely to result from a dysfunctional interaction between components of the intestinal microbiota and an abnormal innate immune system and mucosal barrier [1, 2].

Potential roles for numerous bacteria in CD pathogenesis have been investigated [3–5], but none have been shown to be clearly causative. A possible role for *E. coli* has been highlighted by the isolation of a pathogenic subset labelled adherent-invasive *E. coli* (AIEC) from the mucosa in some patients with CD [6, 7]. Studies show that AIEC survive and replicate within macrophages *in vitro* [8, 9] and that *E. coli* can be isolated from lamina propria (LP) macrophages in CD [5, 10, 11]. This has led to the hypothesis that *E. coli* may cross the mucosal barrier and persist within LP macrophages as a stimulus for chronic inflammation in CD. Of note, most studies report that AIEC are isolated in less than half of CD cases [6, 7] and other less pathogenic *E. coli* can also be recovered by intracellular culture in CD [6, 12]. In addition, recent data demonstrates that macrophages may be dysfunctional in CD [1], and so it is likely that both bacterial and host factors contribute to intra-macrophage bacterial persistence in CD.

Human macrophage phenotypes are heterogeneous and plastic [13, 14]. One broad classification distinguishes M1 inflammatory macrophages, which express high IL-12, iNOS and low IL-10, from M2 regulatory macrophages which express high IL-10 and low IL-12 [14]. In diseases such as atherosclerosis and malignancy, these polarised macrophage phenotypes make particular contributions to pathogenesis [14]. Resident lamina propria macrophages in health are characterised by inflammatory anergy but enhanced phagocytic and microbicidal capacity [15]. In CD, lamina propria macrophages are more numerous, probably due to recruitment of CD14+ monocytes, and have greater pro-inflammatory cytokine expression [16]. However, the roles of differing macrophage phenotypes in CD pathogenesis are incompletely understood.

The aim of this study was to characterize macrophage phenotypes according to *E. coli* carriage in mucosal biopsies from patients with CD, UC and healthy controls. This may provide insight into potential roles for macrophage phenotypes and *E. coli* in CD pathogenesis.

## Methods

### Study participants

Patients with IBD were recruited at routine colonoscopy. Diagnoses of CD and UC were established by conventional criteria. Asymptomatic healthy controls were recruited at surveillance colonoscopy for previous colorectal polyps or a family history of colorectal cancer. Clinical and demographic data were collected. All participants provided written consent and the study received ethical approval (REC ref. 07/H0804/78).

### Mucosal biopsies and biopsy processing

Biopsies were taken from the colon in healthy controls and from inflamed +/- uninflamed sites (ileal (CD only) or colonic mucosa (CD and UC)) in patients with IBD. In a subgroup of CD patients, paired inflamed and uninflamed biopsies were taken from the same segment of bowel. Endoscopic severity was graded using the simple endoscopic score for CD (SES-CD) [17] and the modified Baron’s score for UC [18]. Histological severity was graded from a biopsy adjacent to the study biopsy. Biopsies were snap frozen in liquid nitrogen and stored at -80 °C. Samples were removed from storage and orientated onto OCT media. 6 μm frozen sections were cut onto 1 mm PALM membrane slides.

### Immunodetection of CD68+ macrophages and presence or absence of intracellular *E. coli*

**Fig. 1:**
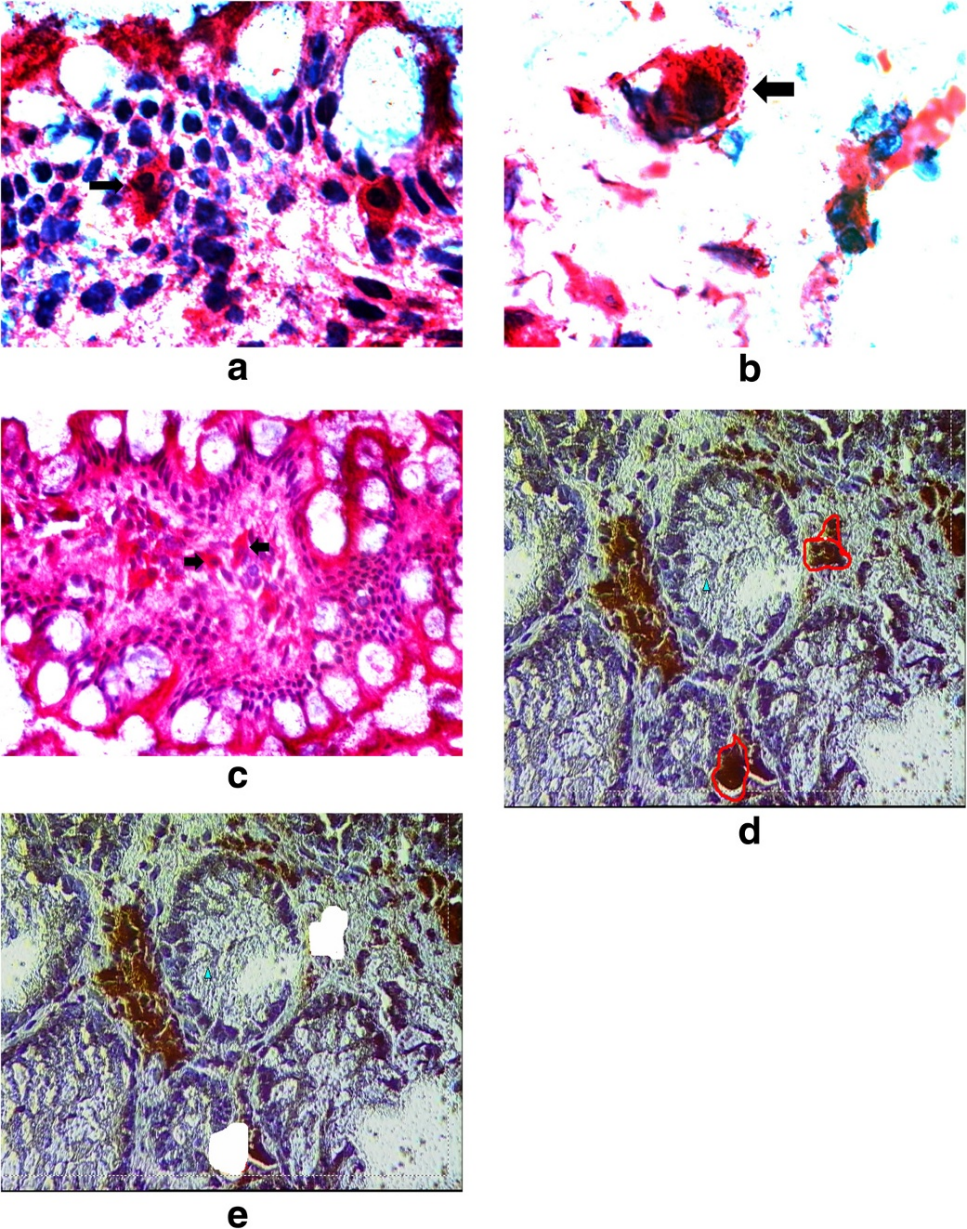
CD68 staining macrophages (red) can be seen to contain *E. coli* ( blue ) in the biopsy from a patient with CD ((**a**) low power (x40), (**b**) high power (x63), but not in biopsies from a healthy control (x20) (**c**). Labelled lamina propria macrophages (**d**) before and (**e**) after laser capture microdissection

LP macrophages with or without *E. coli* co-localisation were identified in mucosal biopsies using previously validated immunolabelled CD68+ [19] and *E. coli* antibodies [20]. A rapid indirect immunostaining protocol was employed for macrophage-specific CD68 (PG M1) to minimize risk of mRNA degradation [21]. CD68 positive cells were detected using a Vectastain ABC-AP kit and a Vector Red chromogenic substrate. Detection of intracellular *E. coli* was achieved by co-staining CD68+ macrophages with an anti-*E. coli* polyclonal antibody and labeled with Vector Blue chromogenic substrate. Staining was visualized using a Zeiss axioplan MOT 400 M microscope. CD68+ cells co-localising with anti-*E. coli* antibody were termed *E. coli*-laden macrophages (Fig. 1a, b). CD68+ cells without anti-*E. coli* antibody co-localisation were termed *E. coli*-unladen macrophages (Fig. 1c).

### Laser capture microdissection (LCM) of macrophages and mRNA extraction

Laser capture was performed using a PALM microsystem. CD68+ only or CD68+/*E. coli* + cells were isolated under direct microscopic visualisation (Fig. 1d, e) and catapulted onto a PALM adhesive cap. 400-500 *E. coli*-unladen and, when present, 400-500 *E. coli*-laden, macrophages were collected per biopsy for sufficient pooled RNA extraction (approximately 35 ng). RNA extraction was performed using the Cells Direct kit (Invitrogen). Cells were incubated with lysis solution at 75 °C for 15 mins. Contents were spun down and treated with DNAse1 for 5 mins. The reaction was stopped by adding 25 mM EDTA and kept on ice. RNA was quantified using a Nanodrop spectrophotometer (Thermofisher) and RNA integrity was assessed using an Agilent 2100 Bioanalyser. The range of RIN values was 8.7 – 9.5 with a cut off of 8.0 for sufficient integrity of RNA [22].

### Primer design

RT-qPCR assays were designed, validated and optimized according to the Minimum Information for Publication of Quantitative Real-Time PCR Experiments (MIQE) guidelines [23]. The mRNA sequences for macrophage activation markers (CD163, iNOS and COX-2) and cytokines (IL-6, IL-8, IL-10, IL-23 and TNFα) (primer details - Table 1) were identified using the Genbank database. Primers were designed using Beacon Designer, version 7.2, selecting for a primer annealing temperature of 55 °C and amplicon length of <100 bp. Primer and amplicon specificity were checked using Primer-BLAST (http://www.ncbi.nlm.nih.gov/tools/primer-blast/) and nucleotide-BLAST (www.ncbi.nlm.nih.gov/BLAST/) respectively. Target secondary structures and primer/template accessibility were assessed using the MFOLD web server (http://www.bioinfo.rpi.edu/applications/mfold) using corrections for ionic conditions of 50 nM Na++ and 3 mM Mg++ and a folding temperature of 55 °C. Lyophilised primers were reconstituted in 1xTE buffer to make a stock solution of 100 μM/μl.

**Table 1:** Oligonucleotide primer pairs used for RT-qPCR analysis

Oligo name	GC %	Sequence
TNFα Sense	50	CGAACATCCAACCTTCCAAAC
TNFα Anti-Sense	42	TGGTGGTCTTGTTGCTTAAAGTTC
iNOS Sense	42	CATCAACAACAATGTGGAGAAAGC
iNOS Anti-sense	55	TCTGCTGCTTGCTGAGGTTG
IL-6 Sense	48	GACAGCCACTCACCTCTTCAG
IL-6 Anti-sense	50	GGAAGGTTCAGGTTGTTTTCTGC
IL-8 Sense	52	CAGCCACTACAAACAGAGCACTG
IL-8 Anti-sense	48	CAAAGGGATGACAAGCAGAAAG
IL-23 Sense	54	GGA CAA CAG TCA GTT CTG CTT GC
IL-23 Antisense	50	GGA GGC TGC GAA GGA TTT TG
IL-10 Sense	55	CCAAGACAACACTACTAAGGCTCCTTT
IL-10 Antisense	48	GCTTCTTATATGCTAGTCAGGTA
COX-2 Sense	59	CTCCTATTATACTAGAGCCCTTCCTC
COX-2 Anti-sense	58	TTTTCCAATCTCATTTGAATCAGG
CD163 Sense	48	AGGGTGATAGAAGAGGCCAACACT
CD163 Anti-sense	50	TTGCACCGGACAAACTTCATGGC

Genbank accession numbers: iNos (L09210), Cox-2 (NM000963), IL-6 (M14584), TNF (M10988), IL-8 (Y00787), IL-10 (M57627), IL-23 (M652720), CD163 (NM0004244)

### RT-qPCR assays

RT-qPCR assays were performed using target-specific primer sequences on a Rotor-Gene 6000, with quantification cycles (Cqs) calculated using Corbett software version 1.7 (build 65). Experiments were carried out in duplicate, and data obtained from replicates yielding a standard deviation of Cq values of <0.5 were used for further analysis.

### Quantification and normalisation of data

mRNA copy numbers were determined from target-specific standard curves of known concentration (10-fold serial dilutions of RNA extracted from fresh frozen tissue biopsy; 100 ng/μl – 1 pg/μl) included with every RT-qPCR run. Cq values were plotted against the logarithm of calculated copy numbers and individual target copy numbers were obtained from the linear regression of the standard curve. Data for each target were normalized against fresh biopsy total RNA and expressed as copies of mRNA/μg total RNA, as recommended [24]. mRNA expression of candidate genes in inflamed CD biopsies was analysed relative to non-inflamed biopsies and controls.

### Statistical analysis

Statistical analysis was performed using SPSS 19.0. Unpaired t-tests and one-way analysis of variance (ANOVA) were used to compare means between 2 groups or greater than 2 groups respectively. Paired t-tests were used for within subject comparisons. Where normality could not be confirmed, non-parametric testing was performed. Fisher’s exact test was used to analyse contingency tables. ANCOVA (ANOVA with covariates) was used to determine the effect of age on expression of cytokines and markers. *P* values < .05 were considered significant.

## Results

### Patients and controls

Data was analysed from 35 patients with CD, 9 with UC and 18 healthy controls. Mean age was greater in healthy controls (49 years) than in patients with CD (37 years) (*P* = .005) but not significantly greater than in patients with UC (39 years) (*P* = .149). Other clinical and demographic details are recorded in Table 2. Biopsy site, and endoscopic and histological severity are recorded in Table 3.

**Table 2:** Clinical and demographic details of study participants

	CD	UC	Healthy controls
Number of participants	35	9	18
Age in years mean (range)	37 (19-65)	39 (27-68)	49 (23-62)
Gender (f, m)	17, 18	3, 6	8, 10
Smoking status			
Never	13	5	10
Current	7	1	3
Ex-	3	2	2
unknown	12	1	3
Disease distribution			
Ileal only	6	-	n/a
Ileo-colonic	9	-	n/a
Colonic only	20	9	n/a
Immunomodulation			
Yes	13	4	0
No	22	5	18
Anti-TNFα therapy			
Yes	2	0	0
No	33	9	18

*CD* Crohn’s disease; *UC* ulcerative colitis; *f* female; *m* male; ex-, ex-smoker; TNFα, tumour necrosis factor-alpha; n/a, not applicable

**Table 3:** Biopsy site and proportion of subjects with E. coli-laden macrophages

Subject group	CD	UC	Healthy controls
Subject numbers	35	9	18
Biopsy site			
rectum	11	9	18
colon (other)	9	-	-
ileum	14	-	-
jejunum	1	-	-
Total	25/35 (71 %) (71 %)((71 %)	1/9 (11 %)	0/18 (0 %)
According to histological severity			
normal	3/7	0/0	0/18
mild	20/26	0/8	-
moderate	2/2	1/1	-
severe	0	0/0	-
According to endoscopic severity score			
CD patients			
SES-CD (biopsied segment)			
0	0/3		
1-2	-		
3	6/11		
4	13/15		
5-7	6/6		
>7	-		
SES-CD (total score)			
0	0/3		
1-2	-		
3-4	8/14		
5-8	9/10		
9-13	7/7		
>13	-		
UC patients			
Modified Baron score			
0		-	
1		0/8	
2		1/1	
3		-	

patient with jejunal CD - SES-CD total not recorded as no colonoscopy

*HC* healthy controls; *UC* ulcerative colitis; *CD* Crohn’s disease

### Prevalence of *E. coli-*laden LP macrophages in CD, UC and healthy controls

**Fig. 2:**
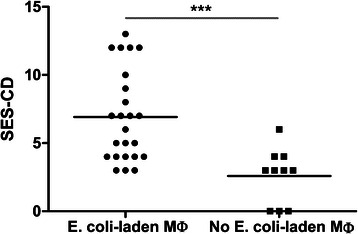
Higher median endoscopic severity score for CD patients with *E. coli*-laden LP macrophages compared with those without *E. coli*-laden LP macrophages at biopsy (*P* < .001, Mann-Whitney test). SES-CD; simplified endoscopic score for Crohn’s disease, Mø; macrophages

*E. coli*-laden macrophages were commonly identified in mucosal biopsies from CD patients (25/35 (71 %)), rarely in UC (1/9 (11 %)) and were not present in any of 18 healthy controls (Table 3). The presence of *E. coli-*laden macrophages in CD correlated with endoscopic severity (*P* < .001) (Fig. 2) but not with other clinical or demographic factors (age, gender, smoking status, disease location, immunomodulation or site of biopsy - data not shown). Six of the 35 CD patients had paired biopsies taken from macroscopically inflamed and uninflamed mucosa. In these 6 patients, *E. coli*-laden LP macrophages were present in 6/6 inflamed and 3/6 uninflamed biopsies respectively.

### Macrophage cytokine and surface marker mRNA expression in CD, UC and healthy controls

#### LP macrophage cytokine mRNA expression in healthy controls is low

**Fig. 3:**
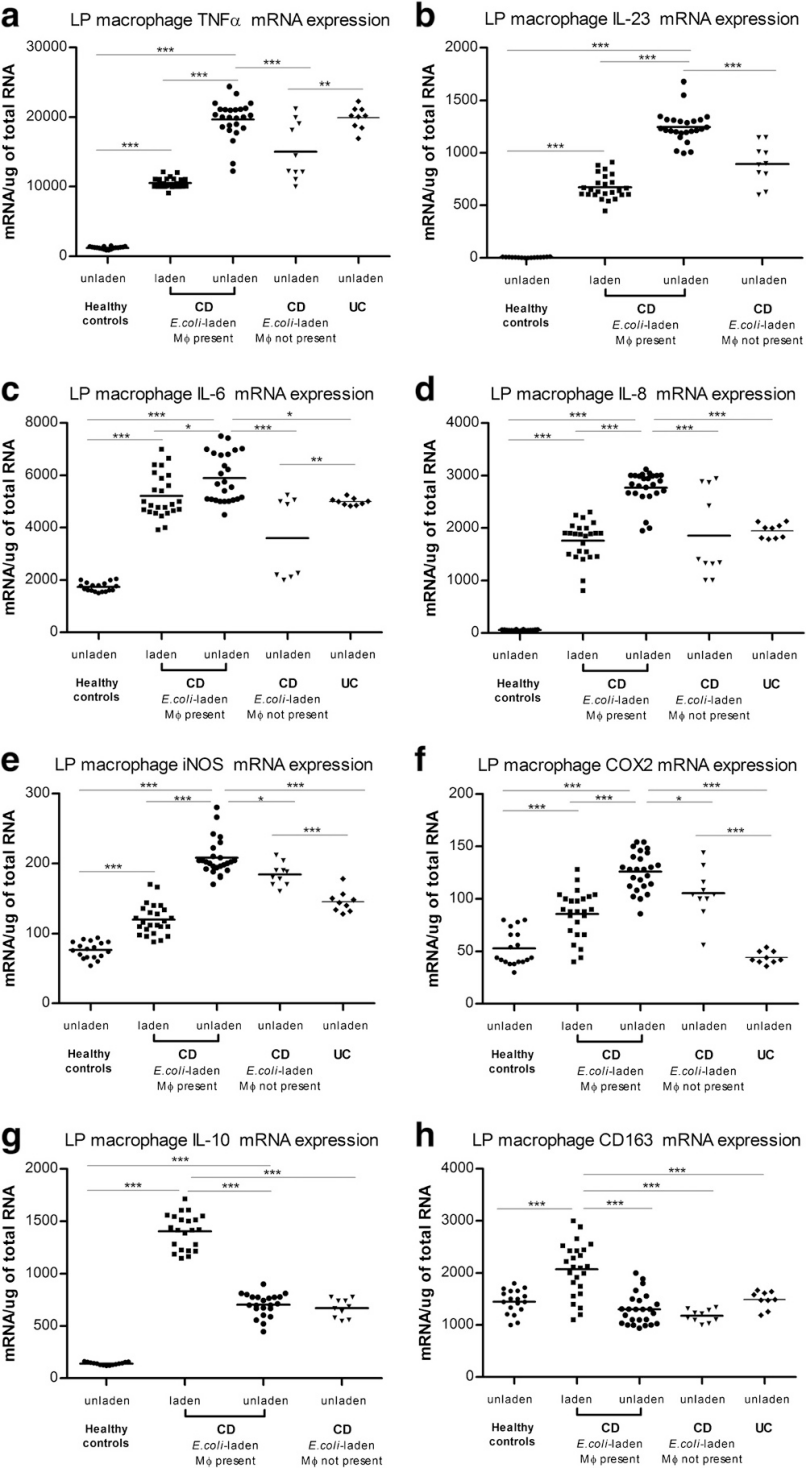
Cytokine and surface marker mRNA levels in healthy controls, CD patients with *E. coli* laden macrophages, CD patients without *E. coli* laden macrophages and UC patients. Fig. 3(**a**) From left to right: Healthy controls (*n* = 18); LP macrophages have low TNFα mRNA expression. Inflamed mucosal biopsies from CD patients with both *E. coli*-laden and unladen macrophages present (*n* = 25); *E. coli*-unladen macrophages had higher mean TNFα mRNA expression (*P* < .001) than *E. coli*-laden macrophages. CD patients without *E. coli*-laden macrophages (*n* = 10); TNFα mRNA expression of *E. coli*-unladen macrophages were lower than in *E. coli*-unladen macrophages from the 25 CD patients in whom both *E. coli*-laden and unladen were present (*P* < .001). UC patients (n = 9); *E. coli*-unladen LP macrophages showed elevated TNFα mRNA levels. Fig 3(**b**) to (**f**): Expression of other proinflammatory cytokines (IL-23, IL-6, IL-8) and iNOS is similar to the pattern of TNFα expression in samples from each subject group. Fig. 3 (**g**) Expression of COX2 is similar to the pattern of TNFα in each group but is lower in UC than in CD. In distinction to the pattern of TNFα expression, Fg. 3 (**g**) and 3(**h**) demonstrate that IL-10 and CD-163 mRNA expression are higher in *E. coli*-laden than *E. coli*-unladen macrophages in CD (for each; *P* < .001). *P < .05, **P < .01, ***P < .001. Comparisons of means made with one-way ANOVA and Games-Howell post-hoc pair-wise comparisons. There was no UC data for IL-23 or IL-10. Only one UC patient had *E. coli*-laden macrophages (cytokine data not shown)

Mean mRNA expression of most cytokines and surface markers (TNFα, IL-23, IL-6, IL-8 & IL-10, iNOS & COX2 (*P* < .001 for each)) was lower in LP macrophages from healthy controls than from *E. coli*-laden or unladen macrophages from inflamed CD mucosa (Fig. 3(a) to (g)). Mean CD163 mRNA expression in LP macrophages from healthy controls was not significantly different to *E. coli*-unladen macrophages (*P* = .173) but lower than *E. coli*-laden CD macrophages (*P* < .001) (Fig. 3(h)).

#### In inflamed CD mucosa, E. coli-unladen macrophages have high proinflammatory cytokine mRNA expression, whereas E. coli-laden macrophages have high IL-10 and CD163 mRNA expression

In inflamed mucosal biopsies from the 25 CD patients in whom both *E. coli*-laden and unladen macrophages were present, *E. coli*-unladen macrophages had higher mean pro-inflammatory cytokines (TNFα, IL-23, IL-6, IL-8) and iNOS expression (Fig. 3(a) – (e), for each; *P* < .001) than *E. coli*-laden macrophages (Fig. 3). *E. coli*-unladen macrophages also expressed higher COX-2 than *E. coli*-laden macrophages (Fig. 3(f)*P* < .001). Conversely, *E. coli*-laden macrophages had higher IL-10 and CD163 expression (Fig. 3(g), (h), for each; *P* < .001) than *E. coli*-unladen macrophages. Macrophage mRNA profiles in CD were not associated with any pattern of disease distribution, biopsy site (ileal or colonic), current IM, anti-TNFα therapy or smoking status (data not shown).

#### E. coli-unladen macrophages are more pro-inflammatory when E. coli-laden macrophages are present

Mean pro-inflammatory cytokine mRNA expression of *E. coli*-unladen macrophages was higher in the 25 CD patients in whom *E. coli*-laden macrophages were also present compared with that in *E. coli*-unladen macrophages in the 10 CD patients’ biopsies in which *E. coli-*laden macrophages were not present (TNFα, IL-23, IL-6, IL-8, iNOS; all *P* < .001) (Fig. 3(a) – (e)).

#### In UC, E. coli-unladen LP macrophages express elevated cytokine mRNA and E. coli-laden macrophages are rare

Only 1/9 biopsies from patients with UC contained *E. coli*-laden LP macrophages. Mean TNFα expression of *E. coli-*unladen macrophages in UC was not significantly different to that in *E. coli*-unladen macrophages in CD (Fig. 3(a)). Other mean cytokine and surface marker expression in UC *E. coli*-unladen macrophages varied from CD as follows: IL-6 & IL-8 not significantly different to CD *E. coli*-laden macrophages; iNOS & CD163, intermediate between CD *E. coli*-laden and unladen; and COX2 not significantly different to healthy controls (IL-23 and IL-10 not measured for UC) (Fig. 3).

#### Macrophage phenotypes in paired inflamed and uninflamed biopsies in CD

**Fig. 4:**
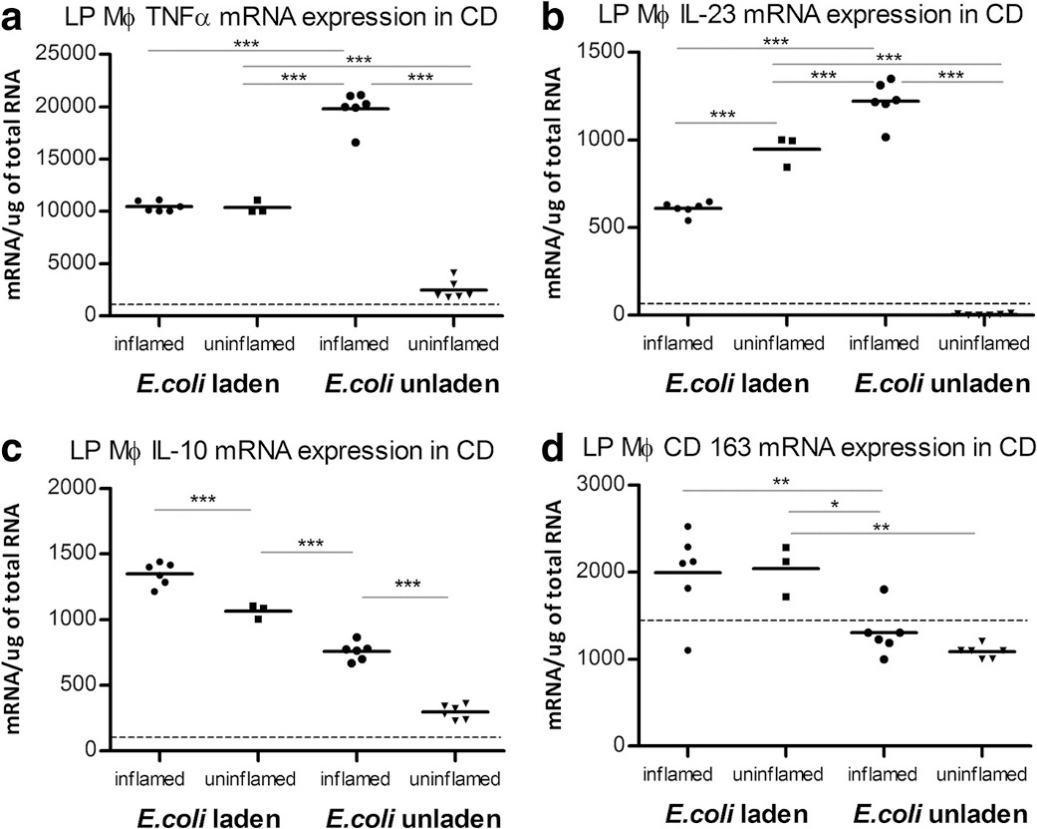
*E. coli*-laden macrophages express a similar phenotype in inflamed and uninflamed tissue high IL-10 (**c**), CD163 (**d**), lower TNFα (**a**) and IL-23 (**b**). *E. coli*-unladen macrophages express a proinflammatory phenotype in inflamed tissue high TNFα (**a**), and IL-23 (**b**), lower IL-10 (**c**), CD163 (**d**) but in uninflamed tissue express cytokine mRNA expression nearer to that of healthy controls (dotted line = healthy control median mRNA expression). MØ = macrophage. *P < .05, **P < .01, ***P < .001. Paired t tests used for within subjects statistical analysis. Presence or absence of inflammation determined by macroscopic appearance at endoscopy

*E. coli-*laden macrophages expressed a similar phenotype in inflamed and uninflamed mucosa (Fig. 4(a) – (d)). Conversely, *E. coli*-unladen macrophages from uninflamed mucosa expressed much lower cytokine and surface marker mRNA levels than in inflamed mucosa (for all *P* < .001 except CD163 *P* = NS) (Fig. 4(a) – (d)). These were at levels closer to, and in some cases, indistinguishable from controls.

## Discussion

In this study, *E. coli*-laden lamina propria macrophages were identified commonly in CD, rarely in UC and not at all in healthy control mucosal biopsies. In CD, there were distinct macrophage phenotypes in relation to the carriage of *E. coli*.

### Intra-macrophage *E. coli* in CD

We found that *E. coli* can be identified within LP macrophages in most CD patients using immunohistochemistry with an anti-*E. coli* specific polyclonal antibody. This concurs with previous studies that demonstrate *E. coli* within LP macrophages in CD using immunohistochemistry [5] and FISH [25] or within granulomas using LCM and nested PCR [10]. In future studies, further confirmation of the presence of *E. coli* within lamina propria macrophages in CD could be achieved using an alternative technique such as 16S rRNA PCR. Additionally, the presence of other bacteria within macrophages could be determined by extracting DNA from laser-captured macrophages, performing bacterial 16S rDNA sequencing and comparing any 16S rDNA sequences present to database reference bacterial sequences [26].

It is possible that the presence of intramacrophage *E. coli* in CD results from successful adherence to and invasion of the mucosa by AIEC, with subsequent survival and replication within LP macrophages. Certainly, *in vitro*, AIEC have been shown to possess properties that might facilitate this process [8, 9]. However, it is also possible that an innate defect of bacterial killing by LP macrophages contributes to *E. coli* persistence within macrophages, a concept which is also supported by the presence of macrophage cytokine defects and mutations in bacterial handling genes in CD [1, 27, 28]. We did not determine whether intra-macrophage *E. coli* were AIEC in our study because these *E. coli* cannot be distinguished morphologically. However, using culture of biopsies with gentamicin protection to isolate intracellular *E. coli* from the mucosa in CD, we have found that only a minority of intracellular isolates have adherent and invasive properties *in vitro* [12].

The lower prevalence (1/9) of *E. coli-*laden macrophages in UC is in keeping with previous reports of lower intramucosal bacteria in UC than CD [6, 29]. Meanwhile, the absence of *E. coli*-laden macrophages in all 18 healthy subjects illustrates that the mucosal immune system prevents bacterial persistence within the LP in health.

### Measurement of macrophage surface marker & cytokine mRNA expression using real time RT-PCR

Real-time RT-PCR can provide a sensitive and accurate measurement of mRNA when performed in accordance with the MIQE guidelines. Normalisation of mRNA data remains a contentious issue. House-keeping genes such as GAPDH and β-Actin have been proposed as being stable appropriate reference genes for normalisation. Prior to initiation of this study, we performed preliminary experiments using GAPDH and β-Actin and found that these reference genes were unstable in these inflamed tissue samples. We also have previously published data demonstrating that internal reference genes may not be appropriate for normalisation of qPCR data for mRNA, especially when derived from tissue biopsies [30, 31]. However, the use of total RNA for normalisation has been demonstrated to be valid [24] and produce quantification results that are biologically relevant [30] as long as certain criteria are met [32, 33]. These are that the RIN is above 8 (which can be considered perfect total RNA for downstream applications [22]) which was confirmed using the Agilent 2100 Bioanalyzer in our study, and the use of small amplicons which minimise the variability caused by RNA degradation. Hence, our decision to use total RNA for normalisation in this study is valid, especially as we are reporting very large and characteristic differences in expression of mRNA for a range of cytokines between Crohn’s disease, healthy controls and UC that are not consistent with chance RNA degradation.

### Characteristic LP macrophages according to *E. coli* carriage in CD

In healthy controls, cytokine and surface marker mRNA expression in macrophages from uninflamed colonic mucosa were low, in keeping with previous data on intestinal macrophages in health [34]. In inflamed CD mucosa, there was clear differentiation of macrophage cytokine and surface marker profiles according to *E. coli* carriage (*E. coli-*unladen; higher proinflammatory cytokines (TNFα, IL-23, IL-6, IL-8) and iNOS, and *E. coli*-laden; higher IL-10 and CD163, both characteristic features of regulatory macrophages). The phenotype of *E. coli*-unladen macrophages in CD is consistent with that of recently recruited CD14+ macrophages, which secrete high TNFα and IL-23 and are more numerous in active CD [16]. It is likely that these activated macrophages contribute significantly to inflammation and recruitment of other pro-inflammatory cells important in CD pathogenesis such as Th17 cells [16]. *E. coli-*unladen macrophages in *uninflamed* mucosa from CD patients with active disease expressed very low cytokine mRNA levels, similar to healthy controls, and are therefore likely to be inactive resident LP macrophages [15].

The observed phenotype (high IL-10, lower TNFα) of *E. coli*-laden macrophages in CD might represent an appropriate regulatory response to microbial encroachment, or may facilitate *E. coli* persistence, and thus contribute to pathogenesis. Supporting the former supposition, the immunoregulatory role of macrophages secreting IL-10 is well documented [35]. However, IL-10 also facilitates intracellular persistence of numerous microorganisms [35], possibly due to inhibition of autophagy [36]. Interestingly, in Whipple’s disease, *Tropheryma Whipplei* accumulate in duodenal macrophages which express a similar phenotype to the *E. coli*-laden macrophages in this study (high IL-10, CD163) which is thought to facilitate their persistence [37].

*E. coli*-laden macrophages were present and also expressed a high IL-10 phenotype in uninflamed mucosa in 3 of the 6 CD patients with active disease in whom paired inflamed/uninflamed biopsies were taken. This raises the possibility that *E. coli* access the mucosa at an early stage of CD pathogenesis rather than as a consequence of a disrupted inflamed mucosa. Of note, the presence of *E. coli-*laden macrophages correlated with endoscopic severity and higher pro-inflammatory cytokine mRNA expression of *E. coli-*unladen macrophages. This highlights further the dilemma of cause and effect as this may either be because the presence of *E. coli* in LP macrophages causes intestinal inflammation or that their presence is merely a consequence of inflammation.

In UC, *E. coli*-unladen macrophage cytokine and surface marker expression were elevated often to similar levels as in CD, however COX-2 mRNA was substantially lower in UC. This may be in keeping with a recent Danish study that reported the association of a COX-2 gene polymorphism (A-1195G variant allele) with UC but not CD [38]. The authors hypothesised that the mutation, which is associated with low COX-2 activity, may lead to increased UC susceptibility because of reduced prostaglandin synthesis as prostaglandins regulate mucosal inflammation. It would be of interest to correlate the prevalence of this mutation with macrophage COX-2 expression in UC in future work.

## Conclusions

This study provides novel insights into the presence of intramacrophage *E. coli* and lamina propria macrophage phenotypes in relation to the presence or absence of intracellular *E. coli* in Crohn’s disease. Further work is required to determine the pathogenic significance of these macrophage subtypes in CD. Clarification of whether *E. coli*-laden macrophages are attempting to ameliorate inflammation or whether they contribute to microbial persistence and disease pathogenesis will also be important. This may lead to therapies aimed at manipulation of macrophage phenotypes [13, 14] and renewed interest in the use of antibiotics that target intracellular *E. coli* for the treatment of CD [39].

## Abbreviations

CD: Crohn’s disease
UC: Ulcerative colitis
HC: Healthy controls
f: female
m: male
ex-: ex-smoker
TNFα: Tumour necrosis factor-alpha
n/a: not applicable
MØ: Macrophage
SES-CD: Simplified endoscopic score for Crohn’s disease
AIEC: Adherent-invasive *E. coli*
